# Is pseudoacetabulum an important factor determining SSTO application in total hip arthroplasty for Crowe IV hips? a retrospective cohort study

**DOI:** 10.1186/s13018-019-1216-8

**Published:** 2019-07-03

**Authors:** Haiwen Peng, Guoqiang Zhang, Chi Xu, Tianhao Wang, Yan Wang

**Affiliations:** 1 Department of Orthopaedic Surgery, Medical School of Chinese People’s Liberation Army, Army Medical University of Chinese People’s Liberation Army, No. 28 Fuxing Road, Haidian District, Beijing, 100853 China; 2 Department of Orthopaedic Surgery, Medical School of Chinese People’s Liberation Army, General Hospital of Chinese People’s Liberation Army, Beijing, China

## Introduction

In terms of developmental dysplasia of the hip (short for DDH), several classification systems have been proposed [1–5]. Most literature is published applicating Crowe’s classification system, because it could be suggestive and instructive for surgery. In 1979, Crowe et al. [1] proposed a four-type system classifying the degree of hip dislocation regarding the ratio of proximal displacement distance of the femoral head in relation to the height of the pelvis or the femoral head, resulting in a calculated coefficient, which converts into one of the four types. Crowe practiced as a hand surgeon after that, so this system was used worldwide and never ameliorative for improvement. In 1987, Eftekhar et al [6] proposed a four-type classification system based on the presence or absence of pseudoacetabulum and overlap between the true acetabulum and the false one. In 1996, Hartofilakidis et al. [4] used the pathology of the dysplastic acetabulum to distinguish between three different types of dysplasia, with subtypes discriminating between a primary and a secondary acetabulum and the relation of the head in relation to these structures. In 2008, Hartofilakidis [7] proposed subtypes on the presence or absence of false acetabulum, and in 2013, he analyzed the clinical outcome of THA based on different subtypes [8]. With the classification of Crowe being increasingly popular, especially in the English literature, it may be the best choice for a classification system with the data available today [9]. In 2016, Ma et al. reported different results between hips of Crowe type IV subtypes on the presence or absence of false acetabulum [10].

High dislocation of hip dysplasia is the most serious type of developmental dysplasia of the hip (short for DDH), which is very difficult to reconstruct the original acetabulum and reset the rotation center to equal both legs and avoid nerve injury after total hip arthroplasty (short for THA). Placing the acetabular component at the true hip center has been shown to provide successful long-term results [11]. However, placement of the acetabular component in the original acetabulum location may result in nerve injury by excessive leg lengthening. This can be addressed by subtrochanteric shortening osteotomy (short for SSTO) first described as a treatment for congenital dislocation of the hip in older children [12], which demands accurate anatomical knowledge and technical expertise for durable results. It was later applied to patients with THA [13]. The shortening subtrochanteric osteotomy (short for SSTO) combined with total hip arthroplasty was a good way to solve the difficulty and showed a good outcome of long-term follow-up. Many authors have reported SSTO with un-cemented THA [14–20]. SSTO procedure was complicated and challenging to surgeons.

In clinical experience, for Crowe IV type developmental dysplasia of the hip, we found that some cases did not need SSTO and could get reduction while others hardly could not get reduction unless SSTO. With understanding and reviewing both the Crowe and Hartofilakidis classification systems, we presumed that the presence or absence of secondary false acetabulum may result in different biomechanical patterns of proximal femur and pelvis, which may lead to differences in the anatomical morphology of the proximal femur, affecting the depth of implantation of the prosthesis, and thus affecting the reduction and necessity of SSTO.

Currently, the S-ROM prosthesis was recognized as the best prosthetic option for severe femoral deformity such as Crowe type IV DDH. This article was designed to investigate the surgical options between the presence and absence of secondary false acetabulum in the treatment of Crowe IV DDH with the S-ROM prosthesis. If difference presents, it is necessary to classify the Crowe IV type DDH into two subtypes depending on if false acetabulum exists or not, which facilitates and instructs surgery.

## Materials and methods

This study was performed with the Investigational Review Board approval. In keeping with the requirements of a retrospective description and review, informed consent was not required. A retrospective cohort study included 32 patients with Crowe type IV DDH underwent THA (34 hip joints) from January 2011 to December 2015. The mean follow-up time was 30 months (from 24 to 70 months).

According to the presence or absence of secondary false acetabulum on the acetabulum side, from January 2011, all subjects were progressively chosen to join this program. There were five males and 27 females with an average age of 42.26 ± 11.20 years (22–68 years), 6 cases of unilateral Crowe type IV DDH, and 19 cases of bilateral Crowe type IV DDH. As of April 2016, all patients had completed follow-up, which included HSS score, X-ray films, and complications. Twenty-eight hips were divided into no false acetabulum group, while 16 hips into the false acetabulum group. In the no false acetabulum group, 14 females and two males had an average age of 39.85 ± 11.46 years old. In the false acetabulum group, 23 females and five males had an average age of 42.01 ± 10.83 year old. The clinical data of age and gender pencertage of the two groups were compared in Table 1. The difference was not statistically significant.

**Table 1:** Different groups depending on if false acetabulum exists or not

Group	No	Gender		Age (mean ± SD, year)
		Female	Male	
False acetabulum	16	14	2	42.01 ± 10.83
No false acetabulum	28	23	5	39.85 ± 11.46
Statistical value		*χ*^2^ = 0.22		*t* = 0.61
*P* value		*P* > 0.05		*P* > 0.05

### Diagnostics, inclusion, and exclusion criteria

Diagnostic criteria are according to the Crowe classification system; Crowe type IV DDH with high dislocation was defined as the ratio of the vertical distance from the head-neck junction to the teardrop line to the diameter of the femoral head > 100% or the ratio of the vertical distance to the height of the pelvis > 20% [1]. Inclusion criteria are strictly in accordance with Crowe type IV. Exclusion criteria are high dislocation of the hip secondary to septic arthritis. The indication for THR in these patients was severe hip pain and impairment of daily activity, regardless of the presence of iliofemoral OA. All medical records were reviewed.

**Fig. 1:**
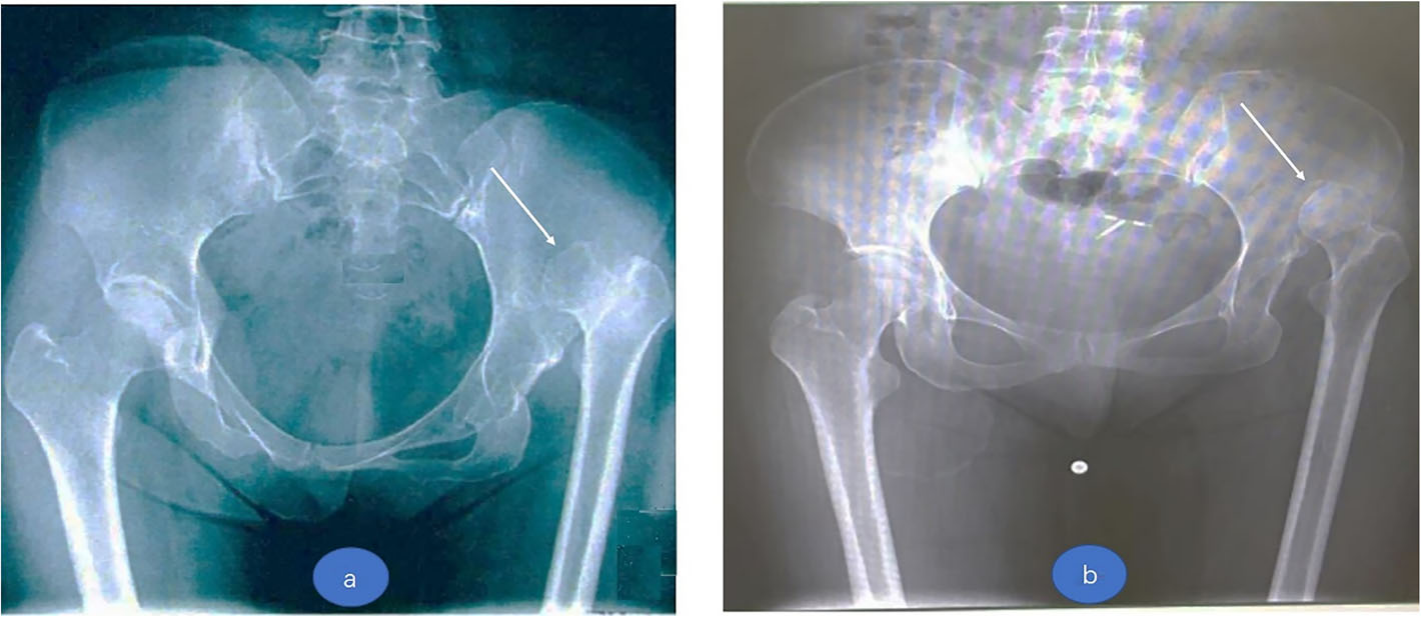
**a** A Crowe IV hip without pseudoacetabulum and dislocated femoral head at posterior ilium place. **b** A Crowe IV hip with pseudoacetabulum and a white line with indication of osteosclerosis emerged at acetabulum side (white arrows point)

The criterion on how we could judge if false acetabulum existed was evaluated on the pelvis plain X-ray. If the femoral head exceeded or reached the lateral ilium wing margin and a white line with indication of osteosclerosis emerged under long-term pressure on plain radiograph, we believed that the femoral head articulated with a false acetabulum. If the femoral head did not reach the lateral ilium wing margin or we could not see any indicating sign of osteosclerosis on plain radiograph, we concluded that no false acetabulum and the femoral head were free floating within the gluteal musculature. See Fig. 1.

## Treatment

The femoral prosthesis of both groups used a modular S-ROM stem ( Depuy, Warsaw, IN, USA), while on acetabular side, Gription Pinnacle cup ( Depuy, Warsaw, IN, USA) was used. The acetabular side was additionally fixed with a screw. The 28-mm-diameter ceramic head was used. Twenty-one hips were applied with SSTO during THA and 23 hips needed not SSTO. All procedures were performed by the same experienced joint surgeon, and the procedure used the posterolateral approach. The external rotation muscle group was separated during operation, the joint capsule was opened, and the femoral head was removed from the base of the femoral neck. The iliopsoas and part of the gluteus maximus are released from the femoral attachments, and the joint capsule was removed to expose the original acetabulum. A ceramic-to-ceramic interface with a 28-mm-diameter head could be used by grinding a 43- or 44-mm hole from a relatively bone stock area in the posterior and lower parts of the original acetabulum. Gription Pinnacle cup with 44-mm diameter could be used.

**Fig. 2:**
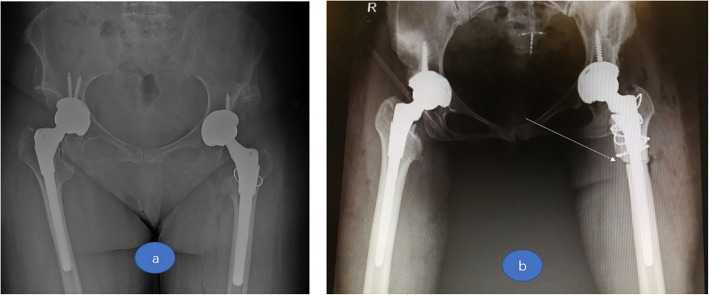
**a** A Crowe IV hip on right side without SSTO in THA. **b** A Crowe IV hip on right side with SSTO in THA (white arrow points)

The proximal femur neck osteotomy was performed at the femoral neck, and the distance between the osteotomy plane and the lesser trochanter plane was determined on the dislocation height. Gradually to the distal but still above the lesser trochanter plane until the femoral stem, trial could be safely inserted. After proximal femur osteotomy, reduction was attempted. If we could get a reduction, true prosthetic components were placed. If reduction was still difficult, SSTO would be chosen (Fig. 2).

### SSTO

The distal femur and proximal medullary cavity were prepared and the S-ROM trial was implanted. The distance between the center of rotation of the femoral head and the center of the acetabulum was measured under traction, and the osteotomy was performed at 10 mm or 20 mm under the small trochanter plane. The bone length that we wanted to remove was the distance between the center of rotation of the femoral head and the center of the acetabulum minus 15 mm (half a femoral head diameter). If a femoral split occurred during the operation, the distal and proximal ends of the femoral trochanter and the osteotomy were tied with steel wire, and the prosthetic test was inserted until the cortex is attached. After the trial implantation, the length and stability of the lower limbs were checked, and the true femoral stem was implanted.

### Postoperative rehabilitation

In order to protect the nerve vascular tissue, the knees were bent 30°. In the morning of the day after surgery, with crutches, patients were encouraged to move down on the ground, which may be partially loaded. At 6 weeks after surgery, the patient could move with full weight. Patients were encouraged to exercise as early as possible to correct pelvic tilt and gait.

### Evaluation after surgery

Patients were followed up 1 month, 3 months, 6 months,12 months, and annually later beyond 1 year after surgery by outpatient review, including physical examination, X-ray, and observation of healing at the osteotomy site and the presence of the prosthesis. Before and after surgery, Harris score of the hip joint was recorded. Postoperative complications such as nerve injury, hip dislocation, and periprosthetic fractures were recorded.

### Statistical processing

All the collected data were statistically processed using SPSS 21st version software (SPSS Inc., Chicago, IL, USA), and the *t* test of quantitative data designed in groups was used to compare the Harris score between the two groups before surgery and during follow-up of patients in both groups. Preoperative and postoperative Harris scores of hip joints were compared by *t* test of paired design quantitative data. By chi-square test, SSTO was compared between the two groups. Chi-square test was used to compare the incidence of complications in the two groups. All statistical tests were performed at 95% bilateral significance level, *P* < 0.05, and the difference was statistically significant.

## Results

SSTO application group with false acetabulum of 16 hips, 3 hips used the SSTO, 18.75%, while in the group without false acetabulum, 8 of the 28 hips were treated with SSTO, accounting for 28.57%. The differences in SSTO used between the two groups were statistically significant (chi-square = 11.33). The proportion of SSTO used in the non-secondary false acetabulum group was higher than that in the secondary acetabulum formation group. See Table 2

**Table 2:** Comparison in SSTO application between different groups

Group	No	SSTO application	
		SSTO	No SSTO
False acetabulum	16	13	3
No false acetabulum	28	8	20
Statistical value		*χ*^2^ = 11.33	
*P* value		*P* < 0.05	

### HSS score evaluation

HSS scores of two groups were compared, and outcomes are shown in Table 3. Preoperative and postoperative (at the last follow-up) Harris scores showed no significant difference in pain, function, or total score between the two groups. Harris scores of all patients after surgery were higher than those before surgery (Table 3).

**Table 3:** Comparison on HSS score

Group	No	Pre-surgery			Post-surgery		
		Pain	Function	Total	Pain	Function	Total
False	16	23.85 ± 10.73	33.35 ± 9.43	57.65 ± 15.24	39.56 ± 4.31	51.23 ± 5.26	88.56 ± 4.79
No	28	27.23 ± 11.46	30.26 ± 11.24	54.23 ± 16.38	41.89 ± 3.29	49.38 ± 4.39	86.43 ± 5.01
*t* value		0.96	0.93	0.68	2.02	1.25	1.38
*P* value		*P* > 0.05	*P* > 0.05	*P* > 0.05	*P* > 0.05	*P* > 0.05	*P* > 0.05

## Discussion

For Crowe type IV DDH, it is difficult to restore the natural rotation center at the original acetabulum place, which has been demonstrated effective and sure of good long-term outcome. The consequence that follows the restoration of the rotation center at original acetabulum place challenges surgeons to avoid nerve injury, and limbs length discrepancy. SSTO procedure is placed on the docket to solve these problems. The necessity of SSTO is determined by varied factors such as dislocation distance, presence or absence of pseudoacetabulum, and so on. Hartofilakidis [6] have previously described two subtypes of high dislocation of the hip, depending on the presence (C1) or absence (C2) of a false acetabulum and reported the different results, especially in survivorship, between C1 and C2 hips regardless of surgery procedure [7]. Taking advantages of the subtype content from the Hartofilakidis classification to Crowe classification since Crowe classification addressed the dislocation distance precisely in ration of proximal displacement distance of the femoral head in relation to the height of the pelvis or the femoral head, the Crowe classification is more effective and efficient as a surgery guideline with consideration of both dislocation distance and presence of pseudoacetabulum. In this study, we determined whether the SSTO application in THA differs between the two aforementioned subtypes of Crowe IV hips. Although there are no other studies in the literature comparing SSTO application in THA in high dislocation subtypes, this outcome seems reasonable, because Crowe IV hips without pseudoacetabulum have more affected femur development and eventual morphology. If pseudoacetabulum exists, the femur has a coverage with compressive stress to develop a relatively approximate normal hip morphology compared with hips without pseudoacetabulum [21]. The presence or absence of secondary acetabulum will lead to different stress patterns of the proximal femur, affecting the development of the proximal femur medullary cavity, and then lead to the difference in anatomical morphology of the proximal femur and the choice of surgical methods. This can answer why some do not need SSTO while others need SSTO in clinical work. Ma et al. [9] reported a similar outcome.

A number of classification systems [1, 4, 6, 22, 23] have been published to grade osteoarthritis secondary to DDH. Crowe et al. [1] defined a four-stage system classifying the degree of dislocation in terms of the percentage of proximal displacement of the femoral head in relation to the height of the pelvis. Hartofilakidis et al. [4, 6] used the pathology of the dysplastic acetabulum to distinguish between three different types of dysplasia, discriminating between a primary and secondary acetabulum and the relation of the head in relation to these structures. Both systems have shown a better reproducibility.

Both Hartofilakidis and Crowe classification systems have deficiencies. The main shortcoming of this Crowe classification is that it cannot reflect the hip pathological changes and consequent biomechanical reconstruction which may have a very long and gradual process. Hartofilakidis et al proposed three different types and later divided low dislocation and high dislocation into two subtypes. The shortcoming of the Hartofilakidis classification system is that it is concerned on only the morphology and insufficient lacks of guidance for surgical treatment since its classification criteria are not precise in form of calculation. We can add these subtypes regarding on the presence or absence of pseudoacetabulum to the Crowe classification system. With combination of these two popular classification systems, it can be more effective and efficient as guidance for DDH treatment.

Several limitations should be considered. First, the study was retrospective in nature and thus was the subject to its inherent limitations and biases. Second, the results were from a single institution and the sample size was not large. However, no previous study has a large sample size because DDH has high morbidity in Asia. Third, the presence or absence of pseudoacetabulum was judged by surgeons without strict criteria, which led to bias. Further prospective studies with larger cohorts are required to validate these results.

## Conclusions

This study reveals that pseudoacetabulum is an important factor determining SSTO application in total hip arthroplasty for Crowe IV hips. We can add these subtypes regarding on the presence or absence of pseudoacetabulum to the Crowe classification system. With combination of these two popular classification systems, it can be more effective and efficient as guidance for DDH treatment option.

## Data Availability

The patients’ data were collected in the Chinese PLA General Hospital. The datasets used and/or analyzed during the current study are available from the corresponding author on reasonable request.

## Abbreviations

DDH: Developmental dysplasia of the hip
SSTO: Subtrochanteric shortening osteotomy
THA: Total hip arthroplasty
